# Fabrication of promising competitive graphene nanocomposite transducer to determine Prucalopride succinate in pharmaceutical formulation and in spiked human biological fluids

**DOI:** 10.1186/s13065-024-01368-z

**Published:** 2025-01-06

**Authors:** Marwa T. Saad, Shereen A. Boltia, Taghreed A. Fattah, Hala E. Zaazaa

**Affiliations:** 1 Pharmaceutical Chemistry Department, Egyptian Drug Authority, Giza, Egypt; 2 Analytical Chemistry Department, Faculty of Pharmacy, Cairo University, Cairo, Egypt

**Keywords:** Calixarene 8, Prucalopride succinate, Graphene nanocomposite, Cation exchanger, Ion Selective electrodes Solid contacted type

## Abstract

The development of a newly fabricated ion-selective electrode (ISE) solid-contacted type for the determination of prucalopride succinate represents a significant advancement in analytical chemistry, particularly in the context of green chemistry principles. The optimization process involved numerous trials to ensure the selection of a cation exchanger and ionophore that offer high sensitivity and selectivity for prucalopride succinate. Through these optimization trials, sodium tetrakis was identified as the most suitable cation exchanger, while calix [8] arene demonstrated the highest affinity towards prucalopride succinate as the ionophore. This careful selection of components ensures accurate and specific detection of prucalopride succinate. To enhance the electroanalytical performance of the ISE, a graphene nanocomposite layer was developed as an ion-electron transducer between the carbon and synthetic polymeric membrane. This graphene-nanocomposite layer improves the overall performance of the ISE, providing a Nernstian slope of 57.249 mV per decade, which aligns with the recommendations of the International Union of Pure and Applied Chemistry (IUPAC). The integration of these components and the utilization of green chemistry principles in the design of the fabricated ISE enable rapid and accurate determination of prucalopride succinate. This innovative approach holds great potential for applications in pharmaceutical analysis and quality control, providing a more sustainable and efficient method for the analysis of prucalopride succinate.

## Introduction

The ion-selective electrode (ISE) is a technique that provides high selectivity based on the composition of its membrane, allowing the absorption of a specific ion. The interaction between the membrane and the solution leads to an ion exchange event or ion transport activity, resulting in an electromotive force (EMF) at the membrane interface [1].

ISE offers several attractive advantages, including its economic and rapid nature. It has a wide concentration range, making it suitable for various applications in industries, environmental monitoring, and clinical diagnostics [2].

However, there are three principal limitations in ISE measurements. First, interference effects from other ions present in the solution can impact the selectivity of the measurement. Second, at high concentrations, the ionic strength effect of the solution can affect the measured activity relative to the exact concentration. Finally, potential drift may occur during a sequence of measurements [3].

Classic liquid-contact ion-selective electrodes (LC-ISEs) are comprised of an ion-selective membrane (ISM) and an internal solution, forming a liquid–contact interface. The theoretical foundation of ISEs relies on the correlation between ion activity and output voltage, as described by the Nernst equation. The electromotive force (EMF) generated by these electrodes is the sum of all the phase boundary potentials [4].

In essence, LC-ISEs operate based on the principle that the potential difference across the ion-selective membrane is proportional to the logarithm of the ion activity in the sample solution. This relationship, established by the Nernst equation, serves as the theoretical basis for the functioning of liquid-contact ion-selective electrodes in measuring and quantifying specific ions in various solutions [5].

The advent of solid contact ion-selective electrodes has provided analysts with a stable sensing platform for designing compact and flexible sensors. However, early iterations of these electrodes encountered potential instability issues, primarily stemming from blocked ion-to-electron transduction and the formation of a water layer. To address these challenges, a hydrophobic conducting polymers layer has been introduced onto the surface of the solid contact [6].

This innovative approach serves as an ion-to-electron transducer, effectively mitigating the formation of a water layer. By doing so, it not only reduces drifts in the sensor but also enhances the overall stability and reproducibility of the potential response. The incorporation of hydrophobic conducting polymers thus represents a crucial advancement, ensuring the reliability and performance of solid contact ion-selective electrodes in various analytical applications.

In the manufacturing of an ion-selective electrode of the liquid-contact type, the first step involves selecting an ideal cation exchanger and a suitable ionophore. This selection is followed by the fabrication of two ion-selective solid-contact sensors, which mainly consist of screen-printed electrodes (SPE). One electrode is plain without a transducer, while the other contains a Graphene Nano Composite (GNC) layer as a transducer in the presence of a polyvinyl chloride (PVC) polymeric membrane. The ionophore and cation exchanger are accurately selected by studying the influence of various molecular recognition ionophores on selectivity. The membrane’s selectivity is enhanced by using a doped membrane with calix [8]arene (CX8) as the ionophore and sodium tetrakis (Na.Tetrakis) as the cation exchanger for both screen-printed electrodes. The effect of incorporating GNC on the performance and selectivity of the electrode is also studied. The GNC electrode improves sensitivity and selectivity in the determination of prucalopride succinate (PRU) without the need for pretreatment steps in pharmaceutical formulations or human biological samples such as plasma and urine. The developed technique is evaluated using the green analytical procedure index (GAPI) approach.

**Fig. 1:**
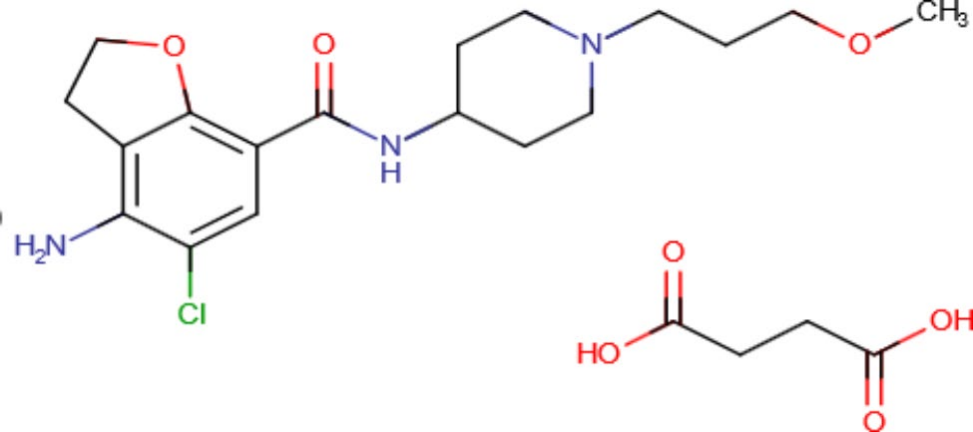
Chemical structure of prucalopride succinate

Prucalopride succinate (PRU), Fig. 1, is a prokinetic agent used to relieve constipation [7]. Various approaches have been reported for the determination of PRU, including chromatographic methods [8–13], spectrophotometric methods [14, 15], spectrofluorimetric methods [16–18] and a voltametric method [19]. To the best of our knowledge, only one potentiometric method has been reported for the determination of prucalopride succinate, which involves the deposition of an ion exchanger (prucalopride succinate-phosphotungstic acid) into a carbon paste fabricated with zeolite particles and dibutyl phthalate [20].

## Experimental

### Apparatus

pH glass electrode (Jenway, Essex, UK) and Jenway digital ion analyzer model 3330 (Essex, UK) with Ag/AgCl double-junction reference electrode (Aldrich Chemical Co. Steinheim, Germany). Bandelin Sonorex, Magnetic stirrer, Rx510S (Budapest, Hungary). Aquatron^®^ water purification unit (A4000D, UK).

### Materials

#### Standard specimens

Prucalopride succinate (PRU) was generously provided by Mash Pharmaceutical industries, Cairo, Egypt and its purity is 99.5% as certified with reported method [21].

#### Pharmaceutical dosage form

Resolor^®^ tablets (batch number is JGL5A00), each single dose contains 2 mg of PRU. Manufactured by Janssen-Cilag and available in Egyptian market.

### Reagents & chemicals

All chemicals are of analytical grade while bi-distilled water was purchased from Aquatron automatic water still (Stuart, UK). Polyvinyl chloride (PVC), sodium tetraphenylborate (NaTPB), calix [4] arene (CX4),4-tert-butyl calix [8] arene (CX8), 2-Hydroxy propyl B-cyclo dextrin and tetrahydrofuran (THF) were gathered from Sigma-Aldrich (Steinheim, Germany). Sodium tetrakis (trifluoromethyl) phenyl borate (MCPE-STFPB) as ion-exchanger and 2-nitrophenyl octyl ether (o-NPOE) was obtained from Alfa Aesar, England. Glacial acetic acid, xylene, sodium hydroxide, boric acid & orthophosphoric acid solution were acquired by El-Nasr (Cairo, Egypt). Graphene nano-platelets (6–8 nm thick×5 micron wide) were purchased from Strem Chemicals Inc. (Newburyport, USA). CH Instruments, Inc. (TX, USA) provided 3 mm diameter carbon screen-printed electrodes (SPE). Urine sample has been collected from a healthy adult female volunteer while plasma samples were collected from biobank of Holding Company for Biological Products and Vaccines in Egypt (VACSERA). A total of 3 plasma samples were used for the study. Each experimental measurement was conducted three times independent replicates to ensure reliability and reproducibility of the data.

#### Preparation of Britton -Robinson buffer pH 4.0

A weight of 2.41 g of boric acid, 2.73 mL of orthophosphoric acid and 2.29 mL of glacial acetic acid were dissolved in 1 L of bi-distilled water. The adjustment of pH to 4 was performed using 2 M sodium hydroxide solution by the aid of pH meter.

#### Preparation of standard solutions

##### Stock standard solution (1 × 10− 2 M) of PRU

A weight of 121.5 mg of PRU bulk powder was transported into a 25-mL volumetric flask then dissolved in an amount regarded to be sufficient using Britton-Robinson buffer of pH 4.0, then that volume was completed up to the mark with the same buffer.

##### Working standard solutions (1 × 10− 7– 1 × 10− 3 M) of PRU

By diluting the stock solution (1 × 10^− 2^ M) of PRU, it was simple to create solutions with different strengths (1 × 10^− 7^ – 1 × 10^− 3^ M). Britton-Robinson buffer (pH 4.0) was utilized to complete the volume. Fresh solutions were provided daily.

## Procedures

### Fabrication of sensors

#### Liquid Contact Electrode (LCE) fabrication

In the preparation of the PVC membranes, six different compositions were used, each with adjusted amounts of the plasticizer 2-nitrophenyl octyl ether (NPOE) and various cation exchangers and ionophores. Sodium tetrakis-[3,5-bis(trifluoromethyl) phenyl] borate (Na.Tetrakis) and sodium tetraphenyl borate (Na.TPB) were used as cation exchangers in the membrane preparation.

For the first three membranes, 190 mg of PVC was added to 10 mg of NaTPB in separate glass rounded Petri dishes with a diameter of 5 cm. Then, 10 mg of one of the three ionophores (CX4, CX8, or β-CD) was added to each dish. The components in each dish were mixed thoroughly. The same procedure was followed for the other three membranes, but Na.TPB was used instead of NaTPB. Afterward, 0.4 mL of NPOE was added to each dish to plasticize the membranes. To ensure homogeneity, 4 mL of tetrahydrofuran (THF) was added to dissolve the membrane components. The dishes were covered and left to evaporate overnight at room temperature, resulting in PVC polymeric master membranes with a thickness not exceeding 0.1 mm.

Rounded disks with an approximate diameter of 8 mm were obtained by cutting the master membrane (LCE) using an appropriate cork borer. THF was used to attach the membrane to a transposable PVC tip, which was snipped into the end of the electrode glass part. As an internal reference solution, equal quantities of 1 × 10^− 4^ M PRU and 1 × 10^− 4^ M KCl (prepared in bi-distilled water) were mixed. An Ag/AgCl wire with a 1-mm diameter was submerged in the internal reference solution to serve as the internal reference electrode.

#### Preparation of 1% graphene nanocomposite (GNC)

The graphene nanocomposite preparation involved the following steps. First, 10.0 mg of graphene nano-platelets powder was dispersed into 1.0 mL of xylene with the aid of ultra sonication for 5 min. This process helped in achieving a uniform dispersion of the graphene in the solvent. Next, 95.0 mg of PVC was dissolved using 0.2 mL of NPOE as a plasticizer. The PVC/NPOE mixture was prepared separately. Then, 3.0 mL of tetrahydrofuran (THF) was added to the PVC/NPOE mixture to create a solution. Subsequently, the graphene dispersion from the first step was combined with the dissolved PVC blend from the previous step. The mixture was subjected to sonication for 10 min to ensure proper dispersion and homogeneity of the graphene within the PVC matrix. The preparation of the graphene nanocomposite was carried out using the solution dispersion method, resulting in a PVC blend with uniformly dispersed graphene nano-platelets, forming the graphene nanocomposite material.

#### Screen Printed Electrode (SPE) fabrication

The membrane mixture containing (Na.Tetrakis) as cation exchanger and CX8 as ionophore was poured in ten microliters on a carbon screen-printed electrode and allowed to evaporate for a whole night at room temperature.

#### Graphene nanocomposite screen printed electrode (GNC/SPE) fabrication

A carbon screen-printed electrode (SPE) was coated with ten microliters of the graphene nanocomposite (GNC) dispersion. The electrode was then allowed to dry at room temperature overnight, ensuring the formation of a stable GNC layer on the electrode surface. After the GNC coating, the fabrication process of the SPE was completed by adding the membrane mixture, as described earlier. The membrane mixture consisted of the appropriate composition of PVC, plasticizer (NPOE), cation exchanger, and ionophore. To prepare the fabricated sensors for use, they were immersed in a 1 × 10^− 4^ M solution of prucalopride succinate (PRU) for 24 h. This step allowed the sensors to equilibrate and establish a stable interaction between the PRU molecules and the membrane components. Following this preconditioning step, the fabricated sensors were ready to be used for the determination of PRU in subsequent analysis.

### Impact of pH

For monitoring the pH impact on the fabricated sensors’ response covering pH variation of 2–11, portions of 2 M HCl and 2 M NaOH solutions were introduced to the working standard solutions of concentration of 1 × 10^− 4^ M and 1 × 10^− 3^ M of PRU. The potential produced for each solution at each pH was recorded.

### Sensors calibration

Aliquots of the previously prepared stock solution and working solutions of prucalopride succinate (PRU) at various concentrations ranging from 1.0 × 10^− 7^ to 1.0 × 10^− 2^ mol/L were transferred into separate 25-mL beakers for the calibration of the sensors. During the calibration process, the solutions in the beakers were stirred using a magnetic stirrer to ensure uniform mixing. Once the sensors reached equilibrium with the PRU solutions, the resulting electromotive force (EMF) readings were recorded. Stable readings within a range of ± 1 mV were observed for all the constructed sensors. To measure the EMF, each electrode was dipped into the respective PRU solution in combination with a double-junction Ag/AgCl reference electrode. Between measurements, the electrodes were cleaned using Britton-Robinson buffer at pH 4 to remove any residue or contamination. The logarithmic concentrations of PRU were plotted against the corresponding observed potentials, resulting in a calibration curve. Regression equations were calculated based on the calibration curve, which allowed for the quantification of PRU concentrations using the measured potentials. When not in use, the sensors were stored inside a beaker containing Britton-Robinson buffer to maintain their stability and prevent any interference or degradation.

### Slope, response Time and operative life’s assessment of the fabricated sensors

The resulting responses acquired from fabricated sensors were accurately studied following IUPAC recommendations.

### Studying the selectivity degree of sensors

To study the influence of interfering substance on designed sensors (which is an important aspect to ensure its selectivity), their selectivity coefficients were calculated following IUPAC guidelines with the aid of this equation:

$$- {\rm{log }}\left( {{{\rm{k}}^{{\rm{pot}}}}_{{\rm{PRU/interferent}}}} \right) = \left( {{\rm{E1 - E2}}} \right){\rm{/S}}$$

E1 is the measured potential of PRU at a concentration of 1 × 10^− 3^ mol/L, E2 is the measured potential of an interference-causing substance at the same concentration of PRU, and S is the slope of the chosen sensor.

### Determination of PRU in Resolor® tablets

To obtain a fine powder, ten Resolor^®^ tablets were accurately weighed and crushed. From this powdered material, 943.8 mg was weighed out to achieve a concentration equivalent to 1 × 10^− 3^ M of prucalopride succinate (PRU). The weighed powder was then transferred and diluted with the appropriate volume of Britton-Robinson buffer at pH 4.0. The resulting mixture was subjected to agitation using a magnetic stirrer for a duration of 30 min. This ensured proper mixing and dissolution of PRU in the buffer solution. For the measurement of PRU concentration, both the double-junction Ag/AgCl reference electrode and the graphene nanocomposite screen-printed electrode (GNC/SPE) were immersed in the prepared solution. The potential generated by the electrodes in the solution was recorded. Using the regression equation derived from the calibration curve, the measured potential value was utilized to calculate the concentration of PRU in the solution.

### Determination of PRU in spiked human plasma

A volume of 0.5 mL of plasma was transferred into a 25 mL volumetric flask. Then, 2.5 mL of the previously prepared PRU solution with a concentration of 1 × 10^− 5^ M was added to the flask. The volume was then made up to the mark with Britton-Robinson buffer at pH 4.0. The resulting solution was transferred to a 25 mL beaker, and the EMF (electromotive force) was measured using the appropriate electrode setup. The measured EMF value was used in conjunction with the regression equation derived from the calibration curve to calculate the concentration of PRU in the plasma sample. The calculated concentration using the regression equation provided excellent recoveries, indicating the accuracy and reliability of the method for determining PRU concentration in plasma samples.

### Determination of PRU in spiked human urine

A volume of 5 mL of human urine was transferred into a 25 mL volumetric flask. Then, 2.5 mL of the previously prepared PRU solution with a concentration of 1 × 10^− 5^ M was added to the flask. The volume was then made up to the mark with Britton-Robinson buffer at pH 4.0. The resulting solution was transferred to a 25 mL beaker, and the emf (electromotive force) was measured using the appropriate electrode setup. The measured EMF value was used in conjunction with the regression equation derived from the calibration curve to calculate the concentration of PRU in the urine sample.

The calculated concentration using the regression equation provided acceptable recoveries, indicating the reliability and suitability of the method for determining the concentration of PRU in human urine samples.

## Results and discussion

Ion selective electrodes (ISEs) offer several advantages as a remarkable analytical tool for quantitative measurements of drugs. These advantages include time efficiency; ISEs provide rapid results, reducing the time required for drug analysis. The measurement process is straightforward and does not involve extensive sample preparation. Cost-effectiveness; ISEs are relatively inexpensive compared to other techniques, making them a cost-effective option for routine drug analysis. The electrodes themselves are affordable, and the method requires fewer resources and reagents especially if it is combined with other inline techniques such as flow injection analysis [22]. Green chemistry approach; ISEs operate without the use of organic solvents, aligning with green chemistry principles. This reduces the environmental impact of the analysis and promotes sustainability. Minimal sample preparation; ISEs eliminate or minimize the need for complex sample pretreatment steps, simplifying the analytical procedure and reducing the likelihood of errors or sample loss. Wide concentration range; ISEs can measure drug concentrations over a broad range, accommodating both low detection limits and high concentrations. This versatility allows for the analysis of various sample types with different concentration levels. Low detection limits; ISEs can achieve low detection limits, enabling the sensitive quantification of drugs even at trace levels. This high sensitivity is beneficial for applications that require precise measurements in samples with low drug concentrations. In summary, the use of ion selective electrodes in drug analysis offers advantages such as time efficiency, cost-effectiveness, adherence to green chemistry principles, minimal sample preparation, wide concentration range, and low detection limits. These benefits make ISEs a superior choice for quantitative measurements of drugs.

Traditional liquid contact ISEs, which rely on internal filling solutions, face drawbacks such as evaporation and handling difficulty due to liquid contact. In response to the need for stable and reproducible ISEs with a long-life span, solid-state ISEs were developed, utilizing substrates like conventional coated-wire electrodes and modified glassy carbon or metallic electrodes. However, all-solid-state ISEs encounter limitations such as the development of interior water films and E_o_ irreproducibility [19]. To address these issues, ion-to-electron transducers like conducting polymers, carbon nanotubes, carbon black, and graphene were introduced. In this type of ion-to-electron transduction, the conversion of charge carriers is not primarily based on the redox activity of the functional material but on the presence of a large double layer capacitance formed at the SC-ISM interface.

Various conducting polymers, including polybenzopyrene, polyacrylate, polythiophene, polyaniline, and polypyrrole, have been proposed as modifications with polymers, leading to improved LODs and signal drifts, but unfortunately without enhancing pH, CO_2_, light, or oxygen sensitivity. Additionally, multiwall carbon nanotubes, single-walled carbon nanotubes, and graphene have been incorporated into ISEs as important ion-to-electron transducers to achieve enhanced electrode stability, higher signal, and elimination of undesired water layers.

Graphene, a two-dimensional carbon allotrope with a one-atom thick planar structure, serves as a core nucleus for various graphitic forms. Its discovery has significantly improved the fabrication of low-cost electrode materials due to its electrical conductivity, carrier mobility, high specific surface area, flexibility, and optical transparency. As a unique macromolecule, graphene exhibits exceptional features such as being impermeable to gases, high thermal conductivity, stiffness, and the ability to tolerate high current densities. These attributes make graphene crucial for applications in nano-electronics, nano-sensors, and nanocomposites.

PRU containing a tertiary amine group, undergoes protonation in an acidic medium, functioning as a cationic center and exchanger when combined with Na.Tetrakis or Na.TPB in PRU-ISM.

### Ion Selective Electrodes (ISEs) performance characteristics

ISEs provide a practical and selective means to measure ions. These sensors are commonly fabricated using a PVC matrix, which serves as a stable support for selective ions in the designed sensors [23]. The polymeric composition of PVC helps maintain the stability of the sensor complexes and prevents their mobilization [24]. However, PVC can be brittle, so careful selection of appropriate plasticizers and adjustment of their ratio with PVC is crucial in this analytical tool. One excellent choice of plasticizer is ortho-nitrophenyl octyl ether (o-NPOE), which enhances the flexibility of the membrane matrix and prevents leaching of the ionophore. Additionally, o-NPOE has a high dielectric constant, facilitating the proper diffusion of PRU throughout the membrane. It is important to note that excessive amounts of o-NPOE can interfere with the selectivity and sensitivity of the sensor, thus requiring optimization of its concentration [25].

Ionophores are low molecular weight compounds that have the ability to dissolve in plasma membranes or intracellular membranes, enabling them to enhance the permeability of specific ions. These compounds play a significant role in various fields such as quality control laboratories, poultry production, and health applications.

One class of ionophores is calix [1]arenes, which are cyclic oligomers formed through the condensation of p-functionalized phenols with formaldehyde [26]. Calix [1]arenes consist of four phenol rings, and their analogues can be synthesized with a higher number of phenol rings using different reaction conditions [27]. In the fabrication of ion-selective membranes, calix [1]arenes are important components due to their basket-shaped cavities, which are rich in electrons. These cavities have the ability to selectively bind and retain specific substances and macromolecules [28].

The electrochemical response of the ion selective electrodes (ISEs) was evaluated by studying the influence of different cation exchangers, ionophores, and pH conditions. Six liquid contact ISEs were fabricated using o-NPOE as a plasticizer, as described in Sect. 3.1.1. Two cation exchangers, Na.Tetrakis and Na.TPB, were utilized along with three ionophores: CX4, CX8, and β-CD. Calibration graphs were generated for each ISE, and the slope and intercept were calculated from the linear part of the graphs. The limit of detection (LOD) was determined based on IUPAC recommendations [29].

When examining the results obtained from the six sensors (as shown in Table 1), it is evident that Sensor 5, which incorporates Na. Tetrakis as a cation exchanger and CX8 as an ionophore, demonstrated the most favorable performance in terms of both slope and limit of detection (LOD). This superior performance can be attributed to several key factors. Firstly, Na. Tetrakis has a higher degree of lipophilicity compared to sodium tetraphenylborate (Na.TPB), allowing it to integrate more effectively into the hydrophobic environment of the ion-selective membrane. This enhances the stability and selectivity of the membrane for cationic species such as Prucalopride (PRU). Additionally, the trifluoromethyl groups in Na. Tetrakis make the borate ion more electron-withdrawing, which improves the selectivity of the ion exchanger toward specific cations like PRU, thereby reducing interference from other ions present in the sample. Moreover, Na. Tetrakis is less soluble in aqueous solutions than Na.TPB, which minimizes the risk of it leaching out of the membrane into the sample solution. This helps maintain the integrity of the membrane and prolongs its functional lifespan. Finally, the structural properties of Na. Tetrakis make it more compatible with the polymeric matrix commonly used in ion-selective membranes, enhancing the overall performance of the membrane in terms of response time, sensitivity, and selectivity.

**Table 1:** Effect of different combinations of ion exchangers and ionophores in the fabricated PVC sensors on the slope, intercept and detection limit of PRU:

Sensor	Ion exchangers	Ionophore	Slope (mV/decade)	Intercept (mV)	Detection limit (Mol/L)
**1**	Na.TPB	CX4.	47.45	257.4	1.0 × 10^− 5^
**2**	Na.TPB	CX8.	48.19	260.33	1.0 × 10^− 5^
**3**	Na.TPB	β-CD.	48.56	263.72	1.0 × 10^− 5^
**4**	Na. Tetrakis	Cx4.	53.44	276.7	2.0 × 10^− 5^
**5**	Na. Tetrakis	CX8.	54.26	280.88	0.5 × 10^− 5^
**6**	Na. Tetrakis	β-CD.	52.79	270.93	0.5 × 10^− 5^
**7**	SPE/ Na. Tetrakis	CX8.	55.68	420.63	0.5 × 10^− 5^
**8**	GNC/SPE Na. Tetrakis	CX8.	57.24	277.59	1.0 × 10^− 6^

In addition, CX8 exhibited the most effective host-guest interaction with PRU, leading to the formation of an inclusion complex. This complex, in turn, facilitated the transfer of polar PRU ions across the hydrophobic membrane. Moreover, the relatively larger cavity of CX8 compared to the other ionophores allowed for robust π-π interactions with PRU, contributing to the formation of a stable and efficient inclusion complex. As a result, Sensor 5 demonstrated enhanced sensitivity and a lower limit of detection, making it the most promising option among the tested sensors. Therefore, this membrane mixture was selected for the fabrication of two other solid contact sensors: the screen printed electrode (SPE) and the graphene nanocomposite screen printed electrode (GNC/SPE).

### Fabricated screen-printed electrodes SPEs with applying of CX8-Na tetrakis membrane

Screen-printed electrodes (SPEs) offer a disposable and cost-effective substrate for various applications. The development of solid contact screen printed ISEs, specifically for the quantification of PRU in quality control (Q.C.) laboratories, has gained significant attention. In this study, the CX8 and Na. tetrakis membrane combination demonstrated the best results in terms of sensitivity and slope (refer to Table 1). The first SPE was doped with the CX8 and Na. tetrakis membrane alone, while the second one was the GNC/SPE, featuring a thin layer of graphene nanocomposite as a transducer, followed by the application of the CX8 and Na. tetrakis membrane.

Graphene, known for its exceptional electrical, mechanical, and thermal properties, as well as its lightweight, flexibility, and chemical inertness [30], was incorporated in this design to achieve excellent recoveries. The proposed design utilizing the graphene nanocomposite as a solid contact ion-to-electron transducer exhibited a wider linear concentration range and a lower limit of detection (LOD) of 1 × 10 ^− 6^ M. The GNC/SPE demonstrated the best electroanalytical performance with a good Nernstian slope (57.249 mV/decade) according to IUPAC recommendations, indicating its selectivity and sensitivity over other electrodes used in the study. Furthermore, the GNC/SPE displayed the capability to determine PRU in dosage forms and biological fluids.

Key factors contributing to the accuracy and precision of the ISE results included well-controlled measuring parameters, electrode selectivity, and the presence of the graphene nanocomposite layer, which effectively minimized potential drift and enhanced repeatability of the GNC/SPE. Notably, the GNC/SPE exhibited minimum drift and complete reversibility without any memory effects. The study presented several innovative results for LCE, SPE, and GNC/SPE in the determination of PRU, as discussed in Table 1.

In comparison with the only published method for determining PRU [20], our proposed potentiometric method demonstrates superiority due to its simplicity and reduced procedural steps. The reported potentiometric method involves multiple steps, including the preparation of ion pairs by separately precipitating PRU with three cation exchangers, followed by the preparation of a zeolite-modified carbon paste electrode.

On the other hand, our potentiometric method not only requires fewer steps but also exhibits enhanced sensitivity, with a linearity range spanning from 1.00 × 10^− 6^ − 1.00 × 10^− 2^ M. This extended linear range enables the determination of PRU in spiked plasma and urine, showcasing the versatility of our method. The combination of simplicity, reduced steps, and heightened sensitivity positions our proposed potentiometric method as a more efficient and effective approach for the accurate determination of PRU compared to the previously published method.

#### Impact of pH

**Fig. 2:**
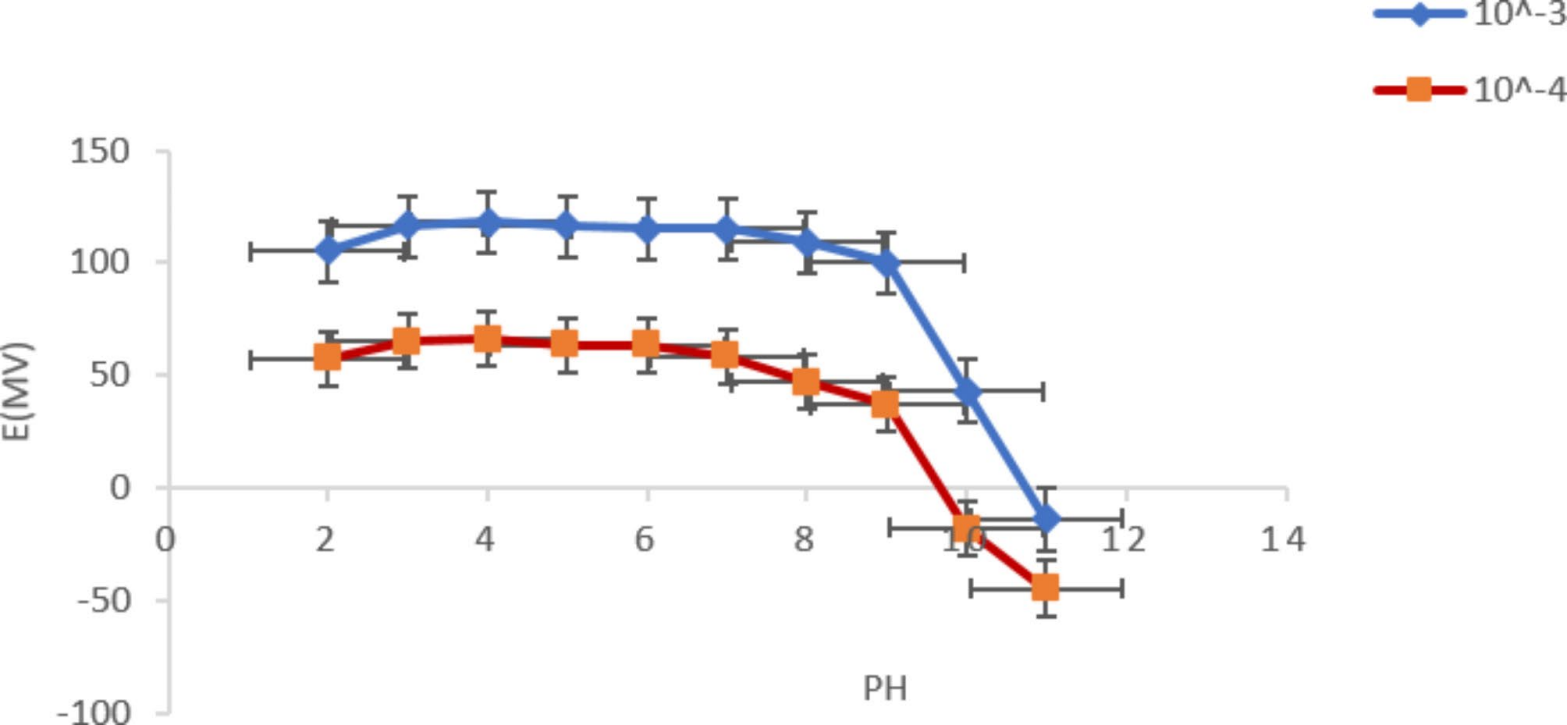
Effect of pH using 1 × 10^− 3^ and 1 × 10^− 4^ mol/L of prucalopride succinate solution with pH range of (2–11)

The impact of pH was validated over a pH range of 2–11 using two concentrations of PRU namely, 1 × 10^− 3^ M and 1 × 10^− 4^ M. the recorded electrodes responses showed relative stability over the range of 3–7 as illustrated in Fig. 2.

Table 2 provides the electrochemical response features and validation parameters of the proposed GNC/SPE sensor. An acceptable Nernstian slope was obtained, demonstrating good sensitivity in terms of LOD. The stability of the sensor was evaluated both daily and within the day by measuring the potential of different concentrations of PRU. The accuracy and precision (repeatability and intermediate precision) of the electrode were calculated, revealing good results for up to 15 to 20 days.

**Table 2:** Electrochemical response characteristics and validation parameters of the proposed sensor for the determination of PRU:

Parameters	GNC/SPE
Slope ^a^ (mV/decade)	57.249 ± 1.7
Intercept ^a^ (mV)	277.59 ± 0.17
Correlation coefficient	0.9998
Linearity range(mol/L)	1.00 × 10^− 6^ − 1.00 × 10^− 2^
Detection limit (mol/L)	1 × 10^− 6^
Response time (seconds)	10
Working pH range	4
Stability (days)	15
Accuracy ^b^ (% recovery ± %R.E.)	100.49 ± 0.57
	100.59 ± 0.57
	101.16 ± 0.57
Repeatability ^c^	0.99
Intermediate precision ^d^	1.15

^a^ average of 2 determinations ± RSD

^b^ results of determination of three concentrations, 1 × 10^− 5^, 1 × 10^− 4^ and 2 × 10^− 5^ mol/L of PRU repeated three times

^c^ RSD of recoveries for the determination of three concentrations (*n* = 9), 1 × 10^− 5^, 1 × 10^− 4^ and 2 × 10^− 5^ mol/L of PRU repeated three times within the day

^d^ RSD of recoveries for the determination of three concentrations (*n* = 9), 1 × 10^− 5^, 1 × 10^− 4^ and 2 × 10^− 5^ mol/L of PRU repeated three times in three different days

**Fig. 3:**
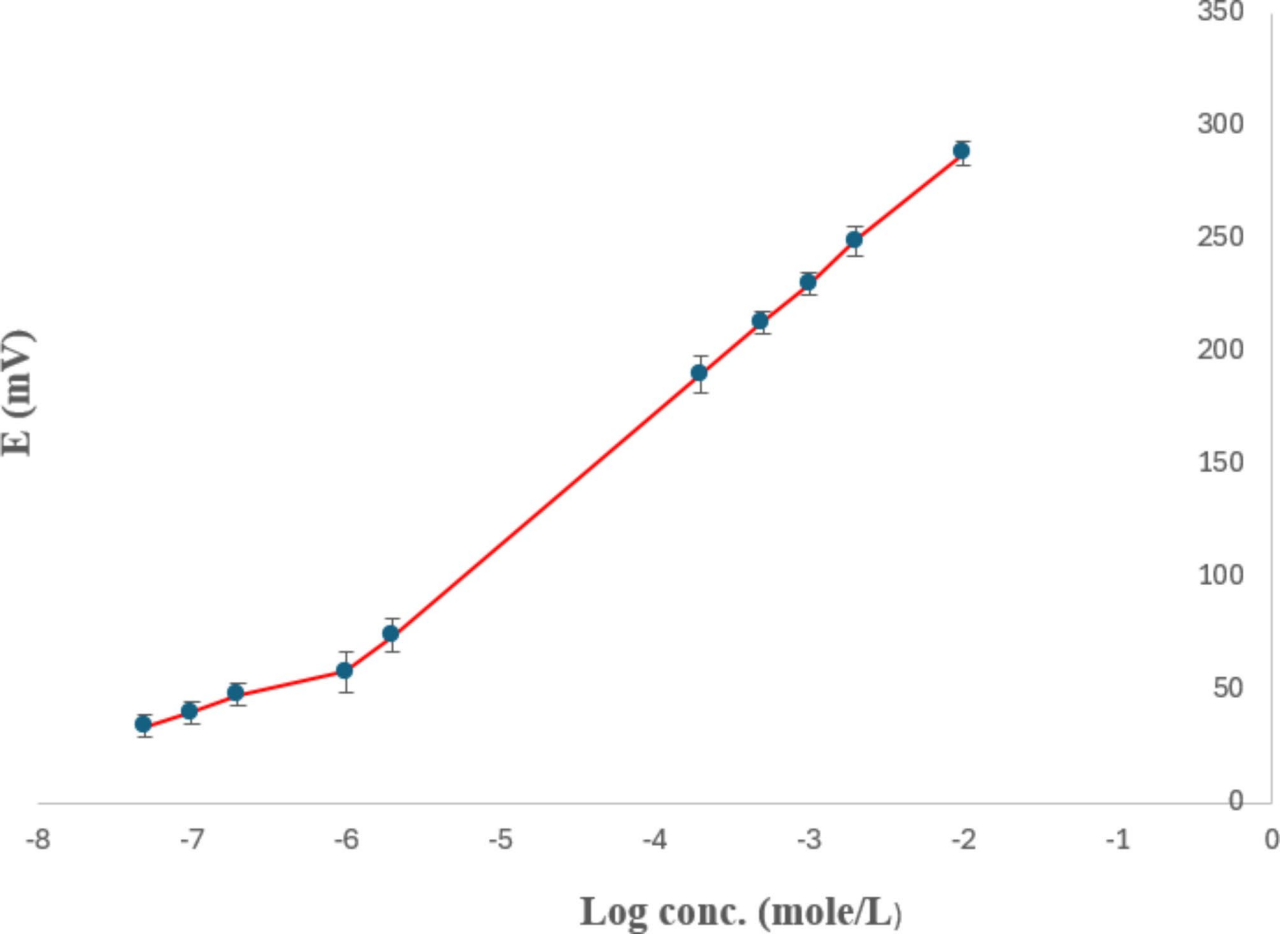
Potentiometric Profile of the potential in mV/− log molar concentration of prucalopride succinate using GNC/SPE

Figure 3 illustrates the potentiometric profile plotted against the logarithm of the molar concentration of PRU using the GNC/SPE. A wide linearity range of 1 × 10^− 6^ to 1 × 10^− 2^ M was obtained.

#### Sensors selectivity

The behavior of PRU towards the GNC/SPE was investigated in the presence of excipients and associated substances using the separate solutions method (SSM) [31]. The selectivity coefficients were determined to evaluate the sensor’s selectivity towards PRU and to assess any potential interference from other substances. The outcomes presented in Table 3 demonstrated that the fabricated sensor exhibited high selectivity towards PRU, with no significant interference from the interfering moieties. This indicates the reliability and accuracy of the sensor in specifically detecting PRU in the presence of other substances.

**Table 3:** Potentiometric selectivity coefficients for the proposed sensor used for the determination of PRU:

Interferent (1 × 10^− 3^ mol/L)	Selectivity coefficient
Maize starch	5.26 × 10^− 4^
Lactose monohydrate	5.77 × 10^− 4^
Talc	5.94 × 10^− 4^
Ammonium sulphate	4.84 × 10^− 4^
Potassium chloride	4.84 × 10^− 4^
Dextrose	5.33 × 10^− 4^
Sodium chloride	5.63 × 10^− 4^
Calcium chloride	6.49 × 10^− 4^
Urea	4.64 × 10^− 4^

#### Potentiometric determination of PRU in Resolor® tablets

The concentration of PRU in Resolor^®^ tablets was determined using the GNC/SPE. The obtained recovery value of 100.15% indicates the excellent detection ability of this sensor for assessing PRU in pharmaceutical dosage forms. Importantly, the use of the GNC/SPE eliminates the need for extraction or pre-treatment steps, simplifying the analysis process. The results are summarized in Table 4, further validating the accuracy and reliability of the sensor in quantifying PRU in Resolor^®^ tablets.

**Table 4:** Determination of PRU in pharmaceutical dosage form by the proposed GNC/SPE sensor:

Preparation	Drug/Claimed potency	Taken	%Found*
Resolor^®^ tablets B.N. JGL5A00	PRU/ 2 mg per tablet	1.0 × 10^− 3^ (mol/L)	100.15 ±1.21

*Average of three determinations

#### Potentiometric determination of PRU in spiked biological fluids

The GNC/SPE, in conjunction with the double-junction Ag/AgCl reference electrode, was employed for the determination of PRU in solutions with a concentration of 1 × 10^− 6^ M. The PRU solutions were spiked with 0.5 mL of plasma and 5 mL of urine separately. The resulting potentials were directly recorded without the requirement of any prior extraction or pretreatment steps for the sample matrices. The exceptional performance of the GNC/SPE was evident in its ability to effectively detect PRU in human biological fluids, as demonstrated in Table 5. The sensor exhibited reliable and accurate measurements in complex sample matrices without the need for additional sample preparation procedures.

**Table 5:** Determination of PRU in spiked human urine & plasma by the proposed GNC/SPE sensor:

Human biological fluid	Added amount of PRU, mol/L	% Recovery *±* RSD
Plasma	1.0 × 10^− 6^	98.20 ± 1.53
Urine	1.0 × 10^− 6^	98.61 ± 1.81

*Average of three determinations

#### Greenness evaluation of proposed ISE method using GAPI approach

The analytical GAPI approach, proposed by Plotka-Wasylka [32], offers a robust methodology for the evaluation and quantification of various environmental factors throughout the analytical procedure. It introduces the concept of the green analytical procedure index (GAPI), which combines the advantages of the NEMI and eco-scale tools while considering the specific aspects of the analytical procedure to assess its environmental impact.

**Fig. 4:**
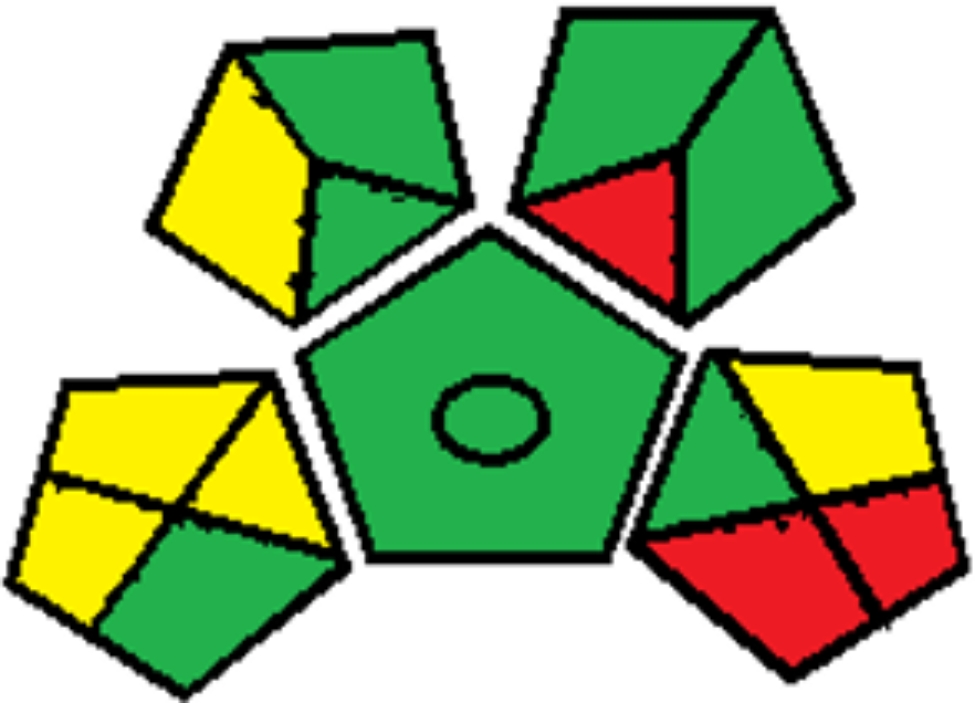
GAPI green profile assessment of the proposed method

The GAPI takes into account 15 parameters associated with different stages of the analytical process, including specimen preparation, solvents and reagents, instruments, waste generation, and waste treatment. Each parameter is represented by a colored pictogram in Fig. 4 of Table 6, indicating its environmental impact as green, yellow, or red.

**Table 6:** Green analytical procedure index parameters description:

Category	Green	Yellow		Red	
**Sample preparation**					
Collection (1)	In-line	On-line or at-line			Off-line
Preservation (2)	None	Chemical or physical			Physico-chemical
Transport (3)	None	Required			-
Storage (4)	None	Under normal conditions			Under special conditions
Type of method: direct or indirect (5)	No sample preparation	Simple procedures, e.g. filtration, decantation			Extraction required
Scale of extraction (6)	Nano-extraction	Micro-extraction			Macro-extraction
Solvents/reagents used (7)	Solvent-free methods	Green solvents/reagents used			Non-green solvents/reagents used
Additional treatments (8)	None	Simple treatments (clean up, solvent removal, etc.)			Advanced treatments (Derivatization, mineralization, etc.)
**Reagent and solvents**					
Amount (9)	< 10 mL (< 10 g)	10–100 mL (10–100 g)			> 100 mL (> 100 g)
Health hazard (10)	Slightly toxic, slight irritant; NFPA health hazard score = 0 or 1.	Moderately toxic; could cause temporary incapacitation; NFPA = 2 or 3.			Serious injury on short-term exposure; known or suspected small animal carcinogen; NFPA = 4.
Safety hazard (11)	Highest NFPA flammability or instability score of 0 or 1. No special hazards.	Highest NFPA flammability or instability score of 2 or 3, or a special hazard is used.			Highest NFPA flammability or instability score of 4.
**Instrumentation**					
Energy (12)	≤ 0.1 kWh per sample	≤ 1.5 kWh per sample		> 1.5 kWh per sample	
Occupational hazard (13)	Hermetic sealing of analytical process	-		Emission of vapours to the atmosphere	
Waste (14)	< 1 mL (< 1 g)	1–10 mL (1–10 g)		> 10 mL (< 10 g)	
Waste treatment (15)	Recycling	Degradation, passivation		No treatment	
**ADITIONAL MARK: QUANTIFICATION**					
Circle in the middle of GAPI: *Procedure for qualification and* *quantification*			No circle in the middle of GAPI: *Procedure only for qualification*		
NFPA: National Fire Protection Association					

By using the GAPI approach, analysts can comprehensively evaluate the environmental sustainability of their analytical techniques and make informed decisions to minimize the environmental footprint of their procedures. The visualization provided by the colored pictograms allows for quick and easy identification of areas that require improvement in terms of environmental impact.

The recorded results obtained using the GNC/SPE in the determination of PRU in dosage form were compared to the results obtained by a previously reported method [21]. The comparison aimed to assess the agreement between the suggested method and the established approach.

Table 7 presents the recorded results from both methods, and it shows that there is no significant difference between the results obtained using the GNC/SPE and the previously reported method. This indicates that the suggested method using the GNC/SPE is comparable in terms of accuracy and precision to the established approach. The similarity in results validates the effectiveness and reliability of the GNC/SPE for the determination of PRU in dosage form.

**Table 7:** Statistical analysis of the results obtained by the proposed ISE method and the reported method for analysis of PRU dosage form

Parameter	Proposed ISE method	Reported HPLC method [21]^*^
**Mean**	99.71	98.56
**S.D.**	1.54	1.38
**n**	3	3
**Variance**	2.39	1.92
F value (19.00) **	1.25	-
Student’s t-test (2.132) **	0.96	-

*The reported method is HPLC method, separation was done on C18 column (KROMASIL 150) using Potassium dihydrogen orthophosphate and Methanol in the ratio (60:40% v/v) at ambient temperature, flow rate was 1 ml/min, injection volume 20 µl

**The values between parenthesis are the corresponding tabulated values of t and F at (*P* = 0.05)

## Conclusion

A novel green approach for the determination of prucalopride succinate in pharmaceutical dosage form and biological fluids was developed using a screen-printed solid-contact ion selective electrode. The incorporation of graphene nanocomposite as the solid contact ion-to-electron transducer enhanced the sensitivity of the electrode, enabling detection of lower concentrations compared to other sensors investigated in this study. The selection of the appropriate ionophore, calix [8]arene, and cation exchanger, Na.tetrakis, played a crucial role in optimizing the microfabrication process and achieving accurate results for prucalopride succinate determination with acceptable precision and accuracy. The optimized graphene nanocomposite screen-printed electrode demonstrated compliance with the guidelines set by the International Union of Pure and Applied Chemistry (IUPAC), exhibiting a good Nernstian slope and a wide linear range. This electrode can effectively be used for the determination of prucalopride succinate in spiked biological fluids, further demonstrating its potential in practical applications.

## Data Availability

Data will be made available on request.
